# Frequency of Seroconversion in Aquaporin‐4 Antibody Testing: Insights From Real‐World Data

**DOI:** 10.1002/acn3.70185

**Published:** 2025-09-17

**Authors:** Tatchaporn Ongphichetmetha, Mengke Du, Nisa Vorasoot, Sean J. Pittock, Jeffrey A. Cohen, Amy Kunchok

**Affiliations:** ^1^ Neurology Cleveland Clinic Neurological Institute, Mellen Center for Multiple Sclerosis Cleveland Ohio USA; ^2^ Siriraj Neuroimmunology Center Mahidol University Siriraj Hospital Bangkok Thailand; ^3^ Department of Neurology and Center for Multiple Sclerosis and Autoimmune Neurology Mayo Clinic College of Medicine Rochester Minnesota USA; ^4^ Division of Neurology, Department of Medicine, Faculty of Medicine Khon Kaen University Khon Kaen Thailand

**Keywords:** aquaporin‐4, demyelination, neuromyelitis optica, NMOSD, seroconversion, serology

## Abstract

Clinicians often repeat aquaporin‐4‐immunoglobulin G (AQP4‐IgG) testing in case of possible seroconversion. Compared with older, less sensitive immunofluorescence assays (IFA), cell‐based assays (CBA) offer higher sensitivity. This study assessed the frequency of seroconversion in a retrospective Cleveland Clinic cohort (2006–2024) of 451 patients with an initial negative AQP4‐IgG result who underwent serial testing. Seroconversion occurred in 4.3% (7/170) of patients initially tested by tissue IFA, but in none (0/263) of those tested by CBA. The lack of AQP4‐IgG seroconversion after a negative CBA, with only rare cases after a negative IFA, suggests that repeat AQP4‐IgG testing is low yield unless prior testing used older methods such as IFA.

## 1 Introduction

The frequency of aquaporin‐4 immunoglobulin G (AQP4‐IgG) seroconversion (negative to positive) in clinical practice is not well understood. Despite this, clinicians often retest seronegative patients out of concern for potential seroconversion, which may add cost and inconvenience for patients. In a laboratory cohort, seroconversion occurred in 0.6% of patients who underwent ≥ 2 AQP4‐IgG tests using cell‐based assay (CBA) methods [1]. This study had a large sample size but limited clinical data and did not examine older test methods. Early AQP4‐IgG testing was done using tissue‐based indirect immunofluorescence assays (IFA), which have low sensitivity (40%–65%) [2, 3, 4, 5]. Later, enzyme‐linked immunosorbent assays (ELISA) offered higher sensitivity (50%–94%) but lower specificity [6]. Currently, the gold standard is CBA, with highest sensitivities (> 85%) reported for live compared with fixed CBA [3, 7].

There are a few studies of seroconversion in clinical cohorts with small sample sizes. A prospective neuromyelitis optica spectrum disorder (NMOSD) cohort study reported a 20.8% (5/24) seroconversion rate [8], while another found 59% (13/22) [9]. We hypothesize that this variation may stem from differences in historical testing methods, including lower‐sensitivity older methods. Other factors influencing false negatives may include the timing relative to disease onset, prior treatments such as immunosuppressive therapies, plasma exchange, or high‐dose steroids, which may reduce titers to undetectable levels [1, 10].

This study aimed to assess the frequency of AQP4‐IgG seroconversion in a large clinical cohort of patients with a suspicion of NMOSD or other central nervous system inflammatory demyelinating diseases, using multiple testing methods.

## 2 Methods

### 2.1 Study Participants and Design

This retrospective cohort study included all patients with ≥ 2 serum AQP4‐IgG tests using various methods and clinical data identified from the Cleveland Clinic electronic records between 2006 and 2024. Patients with a negative result on their first serum AQP4‐IgG test were included. The clinical indication for AQP4‐IgG testing could not be determined retrospectively in all patients; however, at our institution, AQP4‐IgG testing is ordered almost exclusively by neurologists for patients with suspected demyelinating disease.

### 2.2 AQP4‐IgG Testing

Serum was tested by several methods: (1) CBA; (2) IFA; (3) ELISA; and (4) screening with IFA followed by confirmation with ELISA, with positivity reported only when both tests were positive. Seroconversion was defined as a change in serum AQP4‐IgG status from negative to positive. The last available test result was considered final.

In this study, approximately 78% of AQP4‐IgG tests were performed at Mayo Clinic, 20% at Associated Regional and University Pathologists (ARUP), and 2% at Quest and LabCorp combined. Mayo Clinic testing has evolved from earlier methods like IFA and ELISA to live CBA by Fluorescence‐Activated Cell Sorting (FACS) since 2016, with IFA no longer used in isolation. Other laboratories previously used ELISA, and many currently use fixed CBA. Since 2020, all AQP4‐IgG testing ordered at Cleveland Clinic has been completed at Mayo Clinic by live CBA. Including all historical testing methods allowed us to assess seroconversion rates across assays and reflect real‐world clinical practice.

### 2.3 Statistical Analyses

Kaplan–Meier curves examined the probability of seroconversion during the follow‐up period, and the log‐rank test compared the probability of survival based on the initial assay (CBA versus IFA). Differences in clinical and laboratory data between patients with and without seroconversion were analyzed using the bootstrap test or Fisher's exact test, as appropriate. Analyses were performed in R version 4.4.2 [11].

## 3 Results

A total of 451 individuals with ≥ 2 serum AQP4‐IgG tests, whose first test was negative, were included (Figure S1). The mean age was 41 years (standard deviation, SD = 15), and 330 (73%) were female. The median clinical follow‐up period was 6.4 years (interquartile range, IQR 2.95–9.53). The median serological follow‐up period was 1.4 years (IQR 0.45–4.75). The first negative test was performed by CBA in 263 (58.3%) individuals, IFA in 170 (38.7%), ELISA in 4 (0.9%), and both IFA and ELISA in 14 (3.1%) (Table 1). The median number of AQP4‐IgG tests per patient was 2 (range 2–6). Of the 451 individuals, 377 (84%) had 2 tests, and 74 (16%) had ≥ 3 tests.

**TABLE 1:** Comparison of characteristics between seroconverted and the persistently seronegative groups.

Characteristic	All patients (*n* = 451)	Seroconverted group (*n* = 7)	Persistently seronegative group (*n* = 444)	*p* ^a^
Age at the first test, years	41.3 (14.5)	45.8 (12.4)	41.2 (14.6)	0.407
Female, *n* (%)	330 (73.2%)	7 (100%)	323 (72.7%)	0.197
Initial presentation, *n* (%)				
Optic neuropathy	143 (31.7%)	3 (42.9%)	140 (31.5%)	0.845
Unilateral optic neuropathy	127	3	124	
Bilateral optic neuropathy	16	0	16	
Myelopathy	193 (42.8%)	3 (42.9%)	190 (42.8%)	
Short segment myelopathy	130	1	129	
Long segment myelopathy	63	2	61	
Supratentorial syndrome	38 (8.4%)	0 (0%)	38 (8.6%)	
Infratentorial syndrome	36 (8.0%)	1 (14.2%)	35 (7.9%)	
Optic neuropathy with myelopathy	5 (1.1%)	0 (0%)	5 (1.1%)	
Optic neuropathy with infratentorial syndrome	2 (0.4%)	0 (0%)	2 (0.5%)	
Others	34 (7.5%)	0 (0%)	34 (7.7%)	
Final diagnosis, *n* (%)				
Multiple sclerosis	173 (38.4%)	0 (0%)	173 (39.0%)	< 0.001
NMOSD	19 (4.2%)	7 (100%)	12 (2.7%)	
MOGAD	12 (2.7%)	0 (0%)	12 (2.7%)	
ADEM	5 (1.1%)	0 (0%)	5 (1.1%)	
Isolated optic neuritis	57 (12.6%)	0 (0%)	57 (12.8%)	
Isolated myelitis	69 (15.3%)	0 (0%)	69 (15.5%)	
Isolated supratentorial syndrome	5 (1.1%)	0 (0%)	5 (1.1%)	
Isolated infratentorial syndrome	5 (1.1%)	0 (0%)	5 (1.1%)	
Combined IIDs^b^	10 (2.2%)	0 (0%)	10 (2.3%)	
Other inflammatory conditions	35 (7.8%)	0 (0%)	35 (7.9%)	
Other noninflammatory conditions	61 (13.5%)	0 (0%)	61 (13.7%)	
Total follow‐up time, years	6.38 (IQR 2.95–9.53, range 0.15–18.46)	8.7 (IQR 8.2–14.3)	6.2 (IQR 2.9–9.5)	0.031
Times of repeated test	2 (range 2–6)	2 (IQR 2–3)	2 (IQR 2–6)	0.262
Methods of the first test, *n* (%)				
CBA	263 (58.3%)	0 (0%)	263 (59.2%)	0.006
IFA	170 (38.7%)	7 (100%)	163 (36.7%)	
ELISA	4 (0.9%)	0 (0%)	4 (0.9%)	
IFA plus ELISA	14 (3.1%)	0 (0%)	14 (3.2%)	

Abbreviations: ADEM, acute disseminated encephalomyelitis; CBA, cell‐based assays; ELISA, enzyme‐linked immunosorbent assays; IFA, tissue‐based indirect immunofluorescence assays; IIDs, idiopathic inflammatory demyelinating syndromes; IQR, interquartile range; NMOSD, neuromyelitis optica spectrum disorder.

^a^ *p* values were obtained from differences in clinical and laboratory data between patients with and without seroconversion, assessed using the bootstrap test or Fisher's exact test, as appropriate.

^b^ Combined IIDs = the combination of ≥ 2 idiopathic inflammatory demyelinating syndromes, including ADEM, optic neuritis, myelitis, supratentorial syndrome, or infratentorial syndrome.

### 3.1 Seroconversion

Seroconversion by any test type was identified in 7/451 (1.6%) of individuals. After excluding patients tested for very low probability clinical presentations (e.g., dizziness), the seroconversion rate was 7/417 (1.7%). The probability of seroconversion was significantly greater in patients initially tested by IFA 7/170 (4.3%) compared to those initially tested by CBA (0/263 [0%]) (*p* = 0.0045).

**FIGURE 1:**
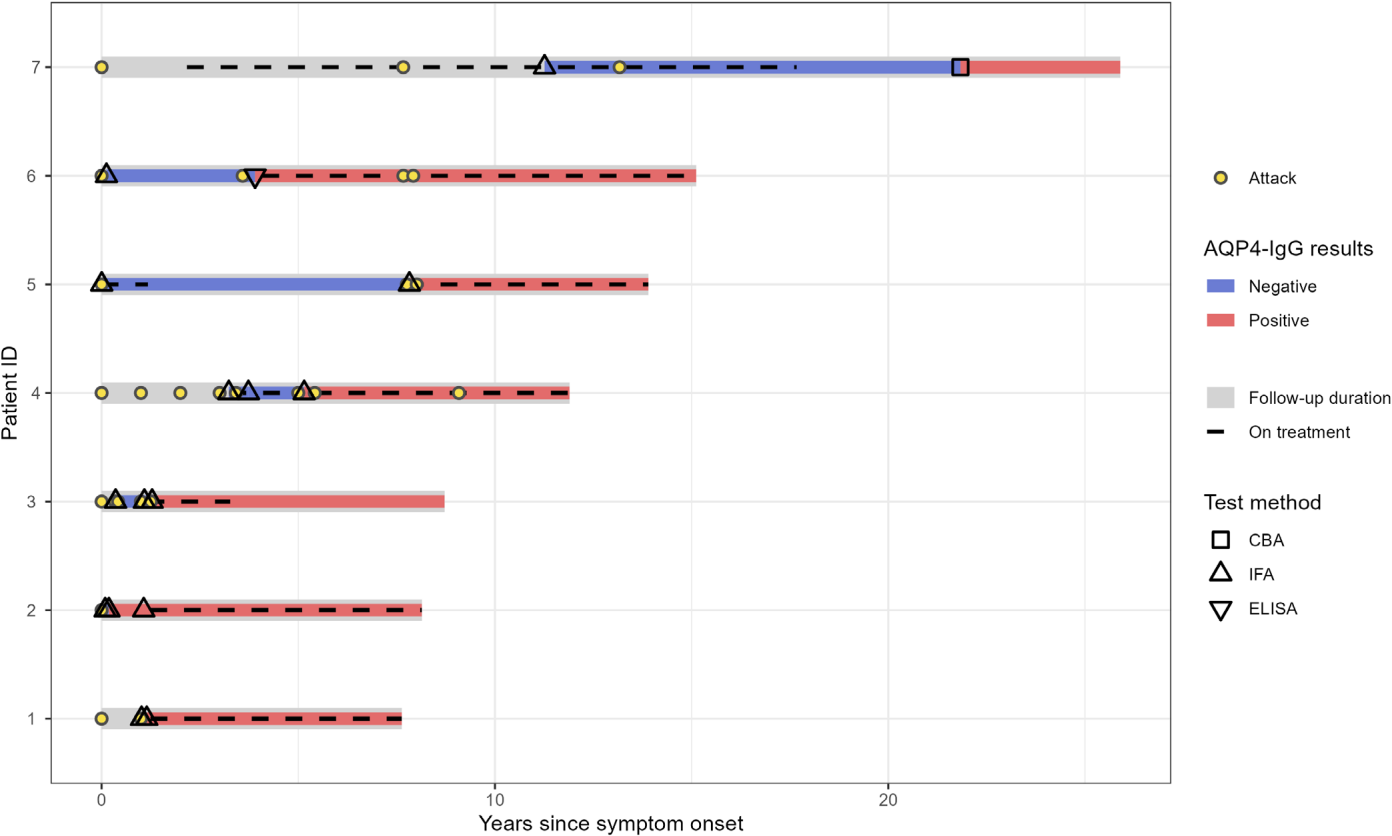
Temporal course of AQP4‐IgG seroconverters: tests, attacks, and therapies. Abbreviations: AQP4‐IgG, aquaporin‐4 immunoglobulin G; CBA, cell‐based assays; ELISA, enzyme‐linked immunosorbent assays; IFA, tissue‐based indirect immunofluorescence assays.

All seven seroconverted patients were initially tested by IFA before 2018, and five returned positive on their second test (3 by IFA, 1 by IFA plus ELISA, and 1 by CBA). Two patients had a negative second IFA test, followed by a positive third IFA test (Figure 1, Table 2).

**TABLE 2:** Clinical and serological features of seroconverters.

Patient number	Sex	Age at first testing, years	First clinical presentation	Number of tests	Duration after first symptom onset, days	Test results (with titer if available)	Test method	Recent or current immunotherapy	Disease course	Tested ±30 days of symptom onset	Final diagnosis
1	F	35	ON	1	908	Negative	IFA	Azathioprine and prednisolone	Relapsing–remitting (recurrent ON and myelitis)	No	AQP4‐IgG+ NMOSD
				2	1090	Negative	IFA	Azathioprine		No	
				3	1608	Positive	IFA	None		No	
2	F	53	ON	1	130	Negative	IFA	None	Relapsing–remitting (recurrent ON and myelitis)	No	AQP4‐IgG+ NMOSD
				2	397	Negative	IFA	None		Yes	
				3	469	Positive	IFA	Rituximab (last dose 2 weeks prior), recent IVMP, and PLEX (1 month prior)		Yes	
3	F	65	Acute myelitis	1	35	Negative	IFA	IVMP (1 dose)	Monophasic	No	AQP4‐IgG+ NMOSD
				2	70	Positive	IFA	Recent IVMP (1 week prior)		No	
				3	393	Positive	IFA	None		No	
4	F	46	Acute myelitis	1	27	Negative	IFA	None	Relapsing–remitting (recurrent ON and myelitis)	Yes	AQP4‐IgG+ NMOSD
				2	2884	Positive (1:40)	IFA	Azathioprine and oral prednisolone		Yes	
5	F	38	ON	1	19	Negative	IFA	IVMP (3 doses)	Relapsing–remitting (recurrent ON)	No	AQP4‐IgG+ NMOSD
				2	1399	Positive (22.8)	IFA + ELISA	Recent IVMP		No	
6	F	52	ON	1	4113	Negative	IFA	Azathioprine	Relapsing–remitting (recurrent ON and myelitis)	No	AQP4‐IgG+ NMOSD
				2	7975	Positive (1:100)	CBA (fixed)	None		No	
7	F	29	Acute brainstem syndrome	1	24	Negative	IFA	None	Relapsing–remitting (recurrent brainstem syndrome)	Yes	AQP4‐IgG+ NMOSD
				2	71	Positive (1:20)	IFA	Recent IVMP (1 month prior) with prednisolone		No	

Abbreviations: AQP4‐IgG, aquaporin‐4; CBA, cell‐based assays; ELISA, enzyme‐linked immunosorbent assays; F, female; IFA, tissue‐based indirect immunofluorescence assays; IVMP, intravenous methylprednisolone; M, male; NMOSD, neuromyelitis optica spectrum disorder; ON, optic neuritis; PLEX, plasma exchange.

### 3.2 Clinical Features of Seroconverters

The mean age of these seven patients was 46 years (SD = 12), and all were female. Of the seven seroconverted individuals, four fulfilled the 2015 Diagnostic Criteria for seronegative NMOSD without AQP4‐IgG (prior to seroconversion) (Figure 1, Table 2). Four patients received immunosuppressants prior to the first negative test, including azathioprine (two patients) and intravenous methylprednisolone (two patients).

**FIGURE 2:**
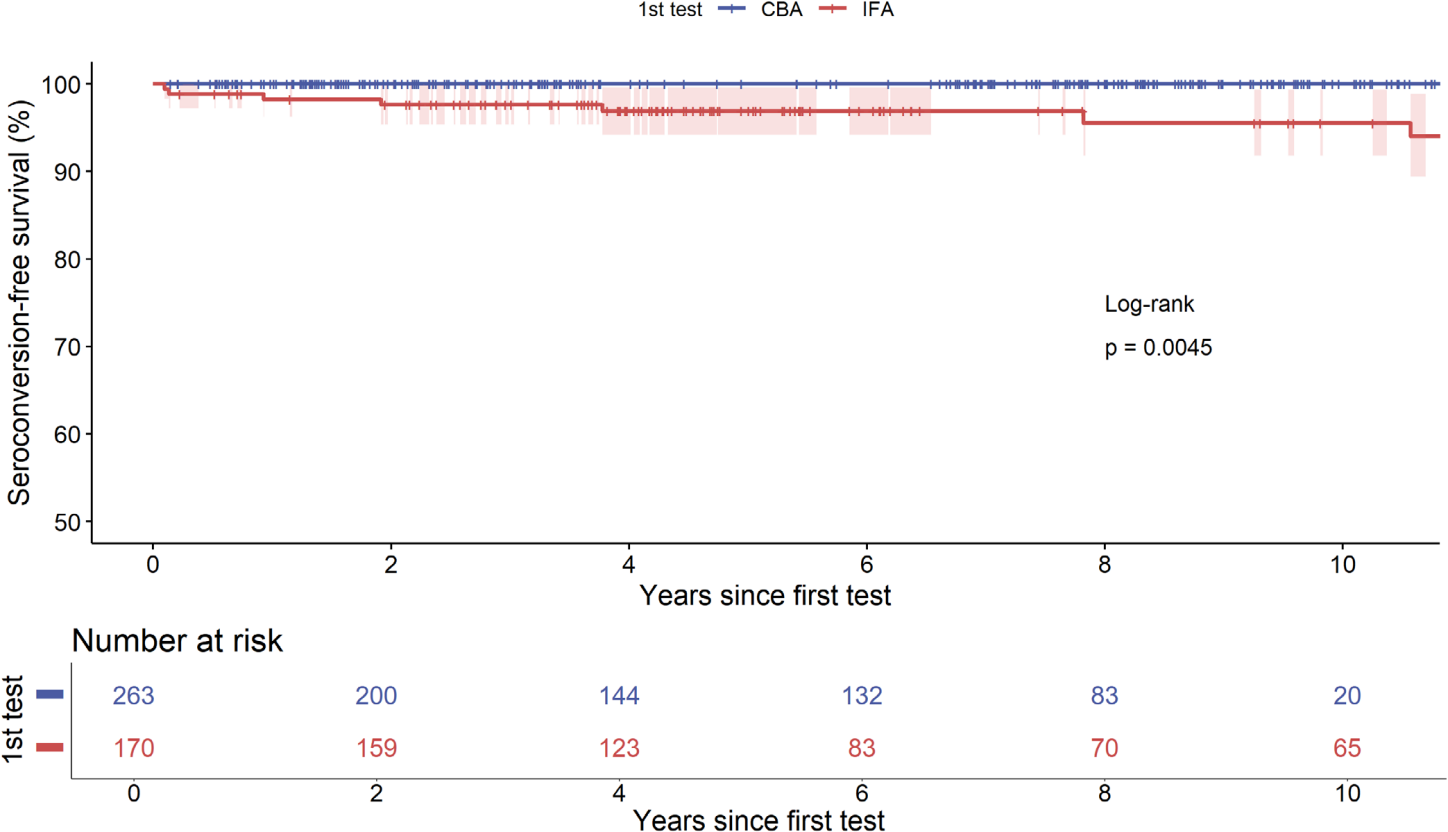
Time to AQP4‐IgG seroconversion after initial negative test with either CBA or IFA. Abbreviations: CBA, cell‐based assays; IFA, tissue‐based indirect immunofluorescence assays.

Seroconversion was most common in patients presenting with myelitis and/or optic neuritis (6/7 [86%]). No patients with a clinical diagnosis of multiple sclerosis (MS) or acute disseminated encephalomyelitis (ADEM) seroconverted to positive. Seroconversion during an attack was observed in two patients. The median time from the first AQP4‐IgG test to seroconversion was 23.3 months (IQR 6.5–70.6) (Figure 2).

## 4 Discussion

In this clinical cohort, AQP4‐IgG seroconversion occurred in 4.3% (7/170) of patients initially tested by IFA, but in 0/263 patients initially tested by CBA. No patient seroconverted after a negative CBA, similar to prior laboratory data (0.6% rate) [1]. These findings suggest that repeat testing is low yield after a negative CBA, but may be warranted for patients previously tested with older assays such as IFA.

The sensitivity of the initial AQP4‐IgG testing method may impact AQP4‐IgG seroconversion. All seven seroconverters were initially tested by IFA (4% of those first tested by IFA). This suggests that the initial negative results could have been false negatives due to the limited sensitivity of IFA. As several studies have reported the low sensitivity of serum AQP4‐IgG testing by IFA [2, 3, 4, 5, 12], patients with an initial IFA test and high clinical suspicion should be considered for retesting with CBA.

Beyond assay sensitivity, disease activity may influence titers of AQP4‐IgG, though prior studies to date have been conflicting regarding this [10, 13]. We observed that two patients had an attack at the time of their seroconversion. Immunosuppressive treatments have also been postulated to influence titers of AQP4‐IgG [1], and we observed that four seroconverters tested negative while receiving azathioprine and corticosteroids, consistent with other reports [1, 14]. However, the role of immunosuppression on serostatus change is not fully understood.

All patients who seroconverted had core clinical features of NMOSD. No patients with a diagnosis of MS or ADEM seroconverted, highlighting both the specificity of AQP4‐IgG and suggesting the low utility of serially testing patients without NMOSD features.

This study has several limitations. First, the initial and repeat testing were determined by treating physicians and not standardized, so not all patients underwent CBA as the final diagnostic test, potentially missing some seroconversions. The specific type of CBA (live vs. fixed) was not specified for all historical tests, though no patient tested by CBA seroconverted. Additionally, the relatively short median serological follow‐up (1.4 years) may have limited the detection of late seroconversion. Strengths of this study include a median clinical follow‐up exceeding 6 years and the availability of comprehensive clinical data.

No AQP4‐IgG seroconversions occurred after a negative CBA in this clinical cohort. Few seroconversions were seen only in patients initially tested with older, less sensitive IFA. Repeat testing with CBA should therefore be reserved for patients with a high clinical suspicion of NMOSD and a prior negative IFA result, as repeating CBA after an initial negative CBA is likely to be low yield.

## Author Contributions

T.O., and A.K., contributed to the conception and design of the manuscript; T.O., M.D., N.V., S.J.P., J.A.C., and A.K. contributed to drafting the manuscript and preparing the figures.

## Conflicts of Interest

T.O., M.D., and N.V. have nothing to report. S.J.P. reported personal fees from Alexion/AstraZeneca (all fees paid to Mayo Clinic), personal fees from Horizon/Amgen (all fees paid to Mayo Clinic), and grants from Roche/Genentech (cytokine testing) outside the submitted work; in addition, Dr. Pittock had a patent for 9891219B2, application 12‐573942, “Methods for Treating Neuromyelitis Optica [NMO] by Administration of Eculizumab to an Individual That is Aquaporin‐4 [AQP4]‐IgG Autoantibody Positive” (he has received royalties). J.A.C. has received personal compensation for consulting for Astoria, Bristol‐Myers Squibb, Convelo, and Viatris, and Chairing a DSMB for Celltrion. A.K. has received personal compensation for consulting for EMD Serono and Alexion.

## Supporting information


**Table S1.** Number of repeat tests after the initial negative result, categorized by initial testing method.
**Figure S1.** Study flow diagram.

## Data Availability

Anonymized data not published can be made available by reasonable request from any qualified investigator, subject to approval by the Cleveland Clinic Institutional Review Board.
