# Supplementary material for: Frequency of Seroconversion in Aquaporin‐4 Antibody Testing: Insights From Real‐World Data
Source: Ann Clin Transl Neurol. 2025 Sep 17;12(10):2158–63. doi: 10.1002/acn3.70185 (PMC12516215; doi:10.1002/acn3.70185)
Supplement: Supplementary file 1 — Table S1. Number of repeat tests after the initial negative result, categorized by initial testing method. Figure S1. Study flow diagram. [file ACN3-12-2158-s001.docx]

**Supplementary Table 1.** Number of repeat tests after the initial negative result, categorized by initial testing method

| **First test method** | **Number of patients** | **Median number of repeat tests following the initial negative result** |
| --- | --- | --- |
| CBA | 163 | 1 (range: 1-4) |
| IFA | 170 | 1 (range: 1-5) |
| ELISA | 4 | 1 (range: NA) |
| IFA+ELISA | 14 | 2 (range: 1-4) |

**Abbreviations:** CBA=cell-based assays; ELISA= enzyme-linked immunosorbent assays; IFA= tissue-based indirect immunofluorescence assays

**Supplementary Figure 1.** Study flow diagram


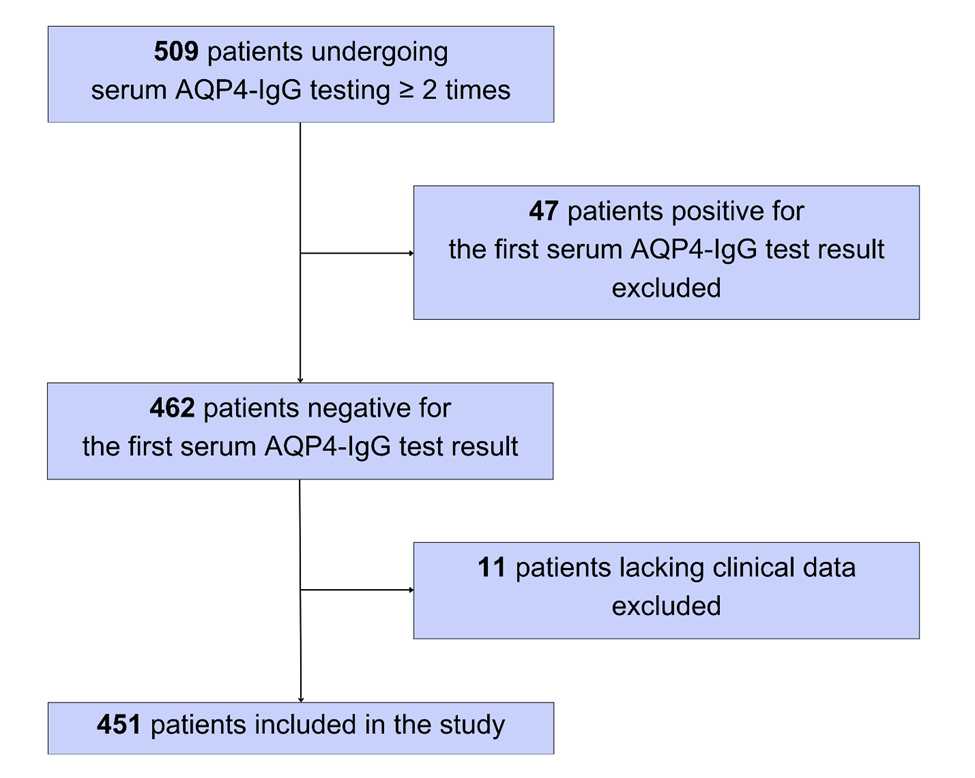


* During the same study period (2006–2024), an additional 238 patients tested positive for serum AQP4-IgG and were not included in this seronegative on first AQP4-IgG test cohort.

**Abbreviations:** AQP4-IgG=aquaporin-4 immunoglobulin G
